# Global prevalence of autism spectrum disorder and its gastrointestinal symptoms: A systematic review and meta-analysis

**DOI:** 10.3389/fpsyt.2022.963102

**Published:** 2022-08-23

**Authors:** Jingyi Wang, Bin Ma, Jingjing Wang, Zeyi Zhang, Ou Chen

**Affiliations:** School of Nursing and Rehabilitation, Cheeloo College of Medicine, Shandong University, Jinan, China

**Keywords:** global, autism spectrum disorder, gastrointestinal symptoms, prevalence, meta-analysis

## Abstract

**Background:**

Autism spectrum disorder (ASD) is a severe public health concern, and Gastrointestinal (GI) symptoms are becoming more common among co-morbidities. The evidence has to be updated depending on differences in different parts of the world. This systematic review and meta-analysis aimed to better understand the existing epidemiological condition and help make health-related decisions.

**Methods:**

Searches in PubMed, Web of Science, Embase databases are limited to 14 March 2022. We reviewed the global prevalence of ASD and the prevalence of GI in people with ASD. Data were extracted by two independent researchers. Literature quality assessment using the National Institutes of Health Study Quality Assessment Tool.

**Results:**

We discovered that the global pooled prevalence of ASD was 98/10,000 (95% confidence interval, 95%CI: 81/10,000–118/10,000, *I*^2^ = 99.99%, *p* < 0.001), with 48.67% (95%CI: 43.50 −53.86, *I*^2^ = 99.51%) of individuals with ASD reporting GI symptoms. Based on the subgroup analyses, we found a higher prevalence of ASD in males (90/10,000, 95%CI: 71/10,000–112/10,000, *I*^2^ = 99.99%) than females (21/10,000, 95%CI: 15/10,000–27/10,000, *I*^2^ = 99.99%). Prevalence of pooling is higher in developing countries (155/10,000, 95% CI: 111/10,000–204/10,000, *I*^2^ = 99.87%) than in developed countries (85/10,000, 95%CI: 67/10,000-105/10,000, *I*^2^ = 99.99%).

**Conclusion:**

The global prevalence of ASD and the prevalence of GI symptoms in ASD are both significant. The prevalence of ASD is much higher in men than in women. Further attention to ASD and its related comorbidities will be required in the future to inform coping strategy adaptation.

## Introduction

Autism spectrum disorder (ASD) is a neurodevelopmental disorder characterized by social communication difficulties and restricted repetitive behaviors that can continue in daily life beginning in early childhood (1). Given the 2–10 fold greater risk of mortality than in the general population (2) and the surge in incidence of the condition (3), ASD is a major problem in the world today. The prevalence of ASD has increased 20–30 fold in the last 40 years worldwide (4). According to research, the prevalence of ASD has risen rapidly worldwide to 1 in 132 (5). Moreover, there is a degree of diversity in the prevalence of ASD among parts of the world. The global prevalence of ASD ranges from 1.4 per 10,000 children in the Arabian Peninsula to 185 per 10,000 children in Asia. In Europe, Sweden has the highest prevalence (115/10,000), while Croatia has the lowest rate (2–3/10,000) (6).

A study showed that over 90% of children with ASD have at least one co-occurring medical condition (7), including gastrointestinal disorders (up to 70%), movement disorders (79%), sleep problems (50–80%) and intellectual disability (45%) (8). In ASD patients, gastrointestinal (GI) problems are common nonneurological symptoms (9), and they have been linked to more severe abnormalities in key ASD areas, such as cognition and behavior (10–13). The prevalence of GI in ASD can range from 9 to 91% due to differences in sample characteristics and survey methods (14). The mechanisms underlying the prevalence of GI symptoms in patients with ASD are inconclusive, but a study has shown that the causes of GI symptoms in patients with ASD may be linked to the gut-brain axis, genetic factors, gut microbiota, and immune responses (15). Children with ASD were two to four times more likely to have functional GI symptoms, the most prevalent of which were constipation, diarrhea, and stomach discomfort, according to a meta-analysis (16).

Given the forementioned, it is crucial to research ASD and GI prevalence. Nonetheless, there are significant differences in previous studies investigating the prevalence of ASD with GI disorders. For example, in Asia, different countries reported contrasting results. In 2017–2018, a study in Riyadh (17) reported that the prevalence of ASD was 2.51%, and a report from Bangladesh (18) stated that the prevalence of ASD was 0.08% in 2016. Similarly, the prevalence of GI with ASD showed disparity. The prevalence of GI in ASD patients reported in the same country during the same publication year varied enormously. For instance, in two studies conducted in the United States in 2021, one found a 34.60% (19) prevalence of GI in ASD, and the other found a 93.18% (20) prevalence. A meta-analysis (16) comparing children with ASD to typically developing children discovered differences in GI symptoms between children with ASD and those without ASD, but the prevalence of GI symptoms was not further investigated, and data on other specific GI symptoms were insufficient. In addition, this review only mentioned the specific situation in the United States and five European countries and did not capture evidence for other geographical areas. The situation regarding mental illness is likely to grow even more grave as a result of the current coronavirus disease 2019 (COVID-19) epidemic. The link between COVID-19 and ASD is currently unknown, although there is evidence that ASD may be a risk factor for heavy COVID-19 (21); thus, more research into the prevalence of ASD is needed. We now need to update the prevalence data for GI in ASD and ASD to provide some references for the health field. The primary objective of this study is to provide an overall estimate of the global prevalence of ASD and the prevalence of GI in ASD patients using the most recent and comprehensive data available to increase public awareness and understanding of ASD and its GI symptoms, and to provide health professionals with references to develop relevant prevention strategies.

## Methods

All analyses were based on previously published studies, so there was no need for ethics approval or patient permission. Our systematic reviews were performed in compliance with the Preferred Reporting Items for Systematic Reviews and Meta-Analyses (PRISMA) (22) and the Cochrane Collaboration Handbook recommendations (23).

### Search strategy

The eligible studies were identified through searches of online databases, including PubMed, Web of Science, and Embase. The literature search was performed up to 14 March 2022. Medical subject headings and keywords were used and combined with Boolean operators: “Autism Spectrum Disorder”, “Autism”, “Prevalence”, and “Gastrointestinal Tract”. Supplementary Table 1 describes the retrieval process in detail.

### Inclusion and exclusion criteria and study selection

Published in peer-reviewed, English-language journals, original studies were considered for inclusion. Our studies incorporate any type of observational study, including cross-sectional studies, cohort studies and case–control studies.

All the eligible studies in this meta-analysis were subjected to the following inclusion criteria: (1) ASD was diagnosed using clinical diagnostic criteria [such as the Diagnostic and Statistical Manual of Mental Disorders [DSM] (24) or International Classification of Disease [ICD] (25) systems, etc.], other validated scales [such as Autism Diagnostic Observation Schedule-Second Edition [ADOS-2] (26), Autism Diagnostic Interview-Revised [ADI-R] (27), Autism Spectrum Screening Questionnaire [ASSQ] (28), Modified Checklist for Autism in Toddlers [M-CHAT] (29)], or a combination of clinical diagnostic criteria and screening tools on ASD, also including parent-reported and specialist diagnosis. GI symptoms were diagnosed by parental questionnaires, ICD diagnosis codes, parent reports, case notes and other means of identifying symptoms. (2) The article contains the total sample size as well as the number of cases, allowing for prevalence estimates. Age, region, and sample size were not restricted. Furthermore, there was no obligatory sample size criterion for selecting papers for the meta-analysis to enlarge the pooled sample. (3) Cross-sectional studies, cohort studies, and case–control studies. The following publications were excluded from this meta-analysis: (1) published case studies, reviews, commentaries, meta-analyses and meeting abstracts; (2) full text was not available; and (3) there were no data available or incorrect. In the event of ambiguity, any differences at each stage were resolved by consensus and the involvement of another experienced expert.

### Data extraction and quality assessment

Using a standardized form, the following data were gathered for all included studies: author, year of publication, country, type of study, sample size, number of cases, percentage of males, age, diagnostic method, and presence of subtype symptoms. Data were extracted independently by two researchers; disputes were resolved through consensus or debate with the third author serving as an arbitrator. If vital information was missing from the original research, it was retrieved by contacting the corresponding authors of potential studies. To assess the quality of each included study, two researchers independently used the 14-item rating scale National Institutes of Health Study Quality Assessment Tool for Observational Cohort and Cross-Sectional Studies and the 12-item rating scale National Institutes of Health Study Quality Assessment Tool for Case–Control Studies (30). The raters' quality ratings were compared, and disputes were addressed through consensus or debate with the third author acting as arbitrator.

### Statistical analysis

For the meta-analyses, we considered papers that used probability sampling methodologies and established disease classifications. In this study, we included the prevalence from the most recently published study if more than one published study reported the prevalence from the same dataset. However, we included data from older research if they provided more details on prevalence statistics than data from newly published studies. Alternatively, if the literature has data from multiple separate studies, we split the data for export (e.g., multiple separate years, data from different cohorts). We used the Stata “metaprop” command to estimate the pooled prevalence (31). The pooled prevalence of estimates was based on the random-effects model (32), which gave an overall estimate across studies weighted by sample size, with the assumption of statistical heterogeneity among studies. To assess statistical heterogeneity, *I*^2^ statistics were utilized, with estimated values of 25, 50, and 75% indicating mild, moderate, and high heterogeneity, respectively (33). A comparison-adjusted funnel plot, which serves as a straightforward visual instrument for detecting the presence of any dominant types of potential bias, such as publication bias, selective reporting, or other biases, was also used to assess potential bias. To determine whether the *P*-values were <0.05, a quantitative Egger's test was used (34). To examine the epidemiology under different subgroup variables according to the variables of interest in this paper, a series of subgroup analyses were conducted. For the total ASD rate, we made the following subgroups: diagnostic method (DSM, ICD, parent-reported and others), regions measured by degree of development (developed countries and developing countries), regions by geography (America, Asia, Europe, Oceania, Africa), year of publication (2001–2010, 2011–2020, 2021–2022), sample size (<10^3^, 10^3^-10^4^, 10^4^-10^5^, 10^5^-10^6^, 10^6^-10^7^, 10^7^-10^8^), sex (male and female), and study type (cohort study and cross-sectional study). For the ASD-GI symptoms rate, we have done the year of publication (2001–2010, 2011–2020, 2021–2022). All the above sequences of analyses were performed in Stata version 15.0.

## Results

### Study selection

**Figure 1:**
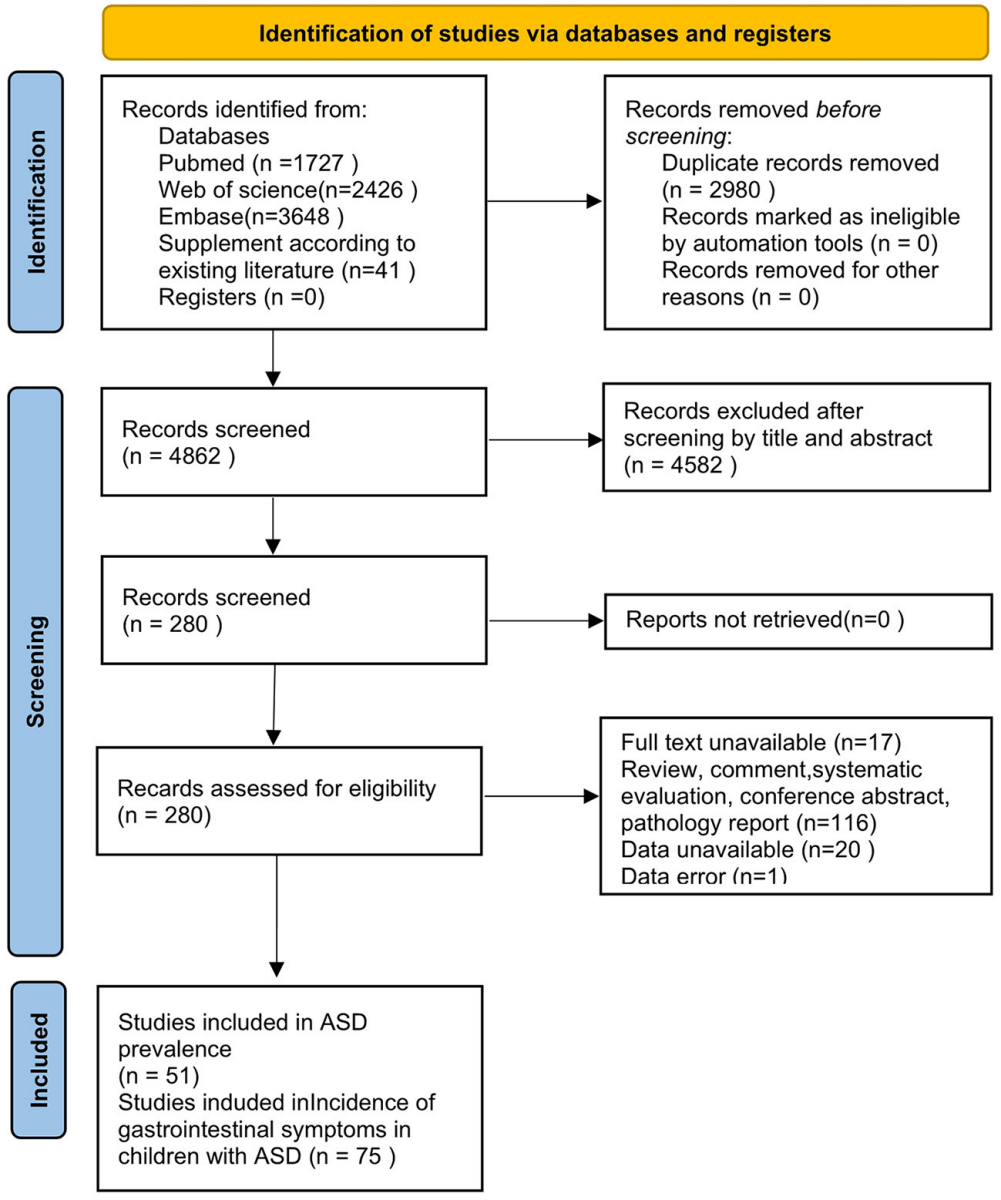
Literature screening flow chart.

We obtained 7,842 studies from the database (41 of which were added manually), of which 2,980 studies were excluded due to duplication and 4,582 articles were removed due to the titles and abstracts, followed by the full-text screening of 280 articles. One hundred fifty-four studies were excluded for various reasons: 17 were not available in full, 116 were nonobservational or original studies (reviews, commentaries, systematic reviews, conference abstracts, case reports), 20 had no available data, and 1 had incorrect data. Finally, we included 126 studies, 51 of which mentioned the prevalence of ASD and 75 of which included the prevalence of GI symptoms in patients with ASD. The details of the research process are shown in Figure 1.

### Quality of the included studies

We assessed the quality of the literature and found that for the 108 cohort and cross-sectional studies, only four studies scored below 8 due to a lack of accurate sample size or impact estimates and failure to account for confounding variables. Of the 34 case–control studies, 8 scored below 6 because the study population was not clearly defined or the control group was not recruited from the same or similar population that caused the cases. Further details of the literature quality assessment can be found in Supplementary Table 2.

### Prevalence of ASD in individuals

Of the 51 studies included, there were 548,413,748 individuals and 1,206,482 people with ASD from all age ranges. The basic information on the prevalence of ASD in all the included studies is described in Supplementary Table 3. The overall pooled prevalence of ASD was 98/10,000 (95% confidence interval, 95% CI: 81/10,000–118/10,000, *I*^2^ = 99.99%, *p* < 0.001), showing significant heterogeneity among studies. The funnel plot was relatively symmetrical, suggesting no significant publication bias ($P_{\text{Egge}{\text{r}}^{\text{′}}\text{stest}}$ = 0.061) (Supplementary Figure 1).

In the subgroup analyses of sex, we found that the pooled prevalence was higher in males (90/10,000, 95%CI: 71/10,000–112/10,000, *I*^2^ = 99.99%) than in females (21/10,000, 95%CI: 15/10,000–27/10,000, *I*^2^ = 99.99%). Depending on the level of development of the countries in which the studies are located, we found differences between developed and developing countries. The prevalence of pooling is higher in developing countries (155/10,000, 95%CI: 111/10,000–204/10,000, *I*^2^ = 99.87%) than in developed countries (85/10,000, 95%CI: 67/10,000–105/10,000, *I*^2^ = 99.99%). In addition, there are 5 geographical regions depending on where the countries are located. Africa (2963/10,000, 95%CI: 2522/10,000–3434/10,000) had the highest total prevalence, followed by Oceania (258/10,000, 95%CI: 156/10,000–384/10,000, *I*^2^ = 95.20%) and the Americas (129/10,000, 95%CI: 106/10,000–155/10,000, *I*^2^ = 99.99%), while Asia (34/10,000, 95%CI: 28/10,000–40/10,000, *I*^2^ = 99.99%) and Europe (69/10,000, 95%CI: 58/10,000–82/10,000, *I*^2^ = 99.62%) had a lower overall prevalence. The pooled prevalence from 2001 to 2010 was 55/10,000(95%CI: 46/10,000–66/10,000, *I*^2^ = 99.29%), the pooled prevalence from 2011 to 2020 was 85/10,000(95%CI: 77/10,000–94/10,000, *I*^2^ = 99.99%), and between 2021 and 2022, the pooled prevalence was 118/10,000(95%CI: 92/10,000–147/10,000, *I*^2^ = 99.98%). Details of subgroups analyses were all presented in Table 1.

**Table 1:**
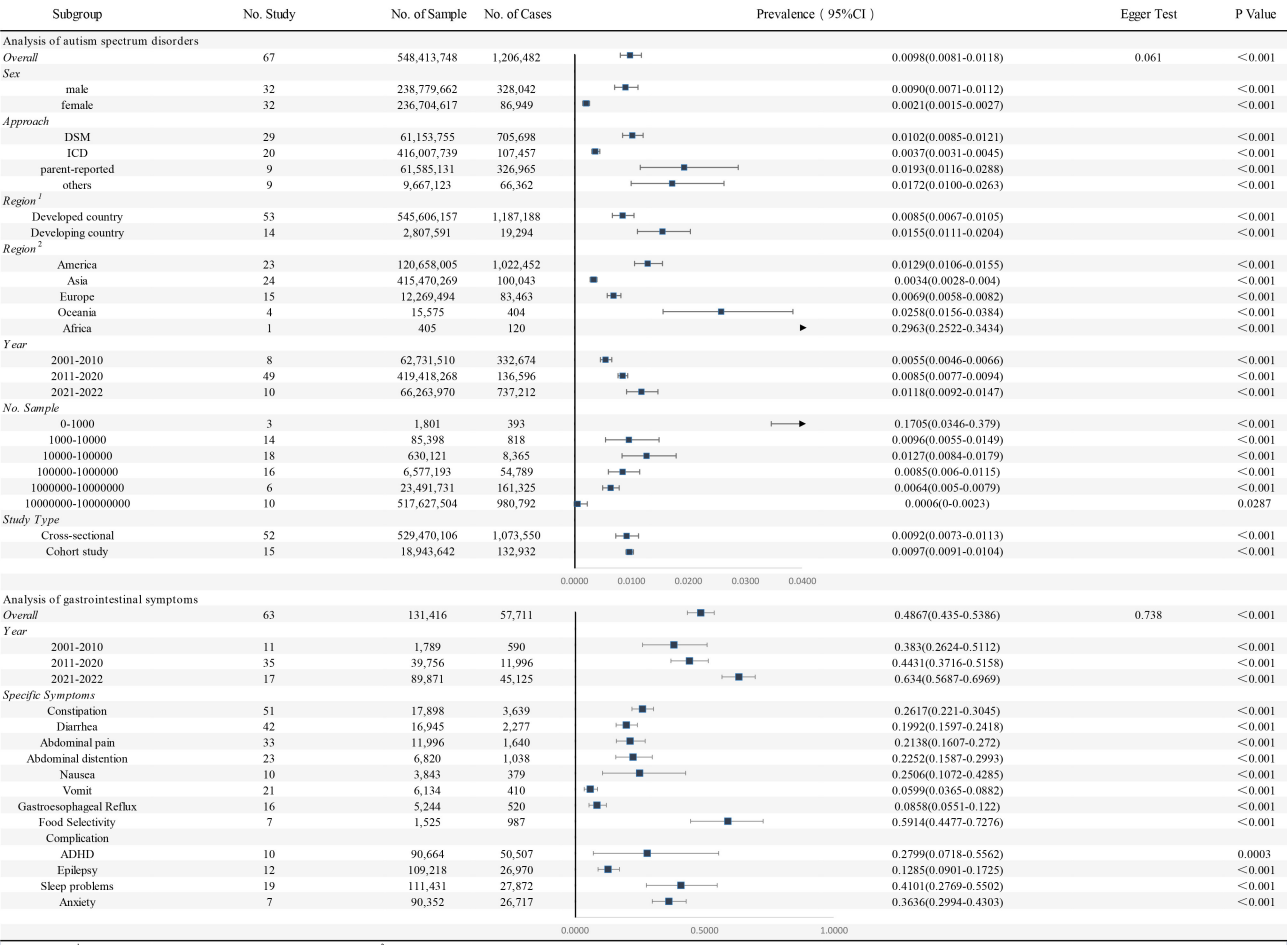
Pooled prevalence of gastrointestinal symptoms in autism spectrum disorders and autism spectrum disorders.

Region^1^ is classified according to developed/developing countries; Region^2^ is based on the region of each country.

➀95%CI:95% confidence interval ➁DSM, Diagnostic and Statistical Manual of Mental Disorders ➂ICD, International Classification of Diseases ➃ADHD, Attention deficit and hyperactivity disorder.

### Prevalence of GI symptoms in individuals with ASD

In total, the 63 studies included 131,416 participants with ASD, of which 57,711 had GI symptoms. Information on specific study characteristics is summarized in Supplementary Table 3. We found that the pooled prevalence of GI symptoms in patients with ASD was 48.67% (95%CI: 43.50–53.86, *I*^2^ = 99.51%). The funnel plot was relatively symmetrical, suggesting no significant publication bias ($P_{\text{Egge}{\text{r}}^{\text{′}}\text{stest}}$ = 0.738) (Supplementary Figure 2).

We observed the occurrence of many specific GI symptoms described in studies. In most of the studies, the following main specific symptoms were listed: constipation (26.17%, 95%CI: 22.10–30.45, *I*^2^ = 97.22%), diarrhea (19.92%, 95%CI: 15.97–24.18, *I*^2^ = 97.47%), abdominal pain (21.38%, 95%CI: 16.07–27.20, *I*^2^ = 97.87%), bloating (22.52%, 95%CI: 15.87–29.93, *I*^2^ = 97.44%), nausea (25.06%, 95%CI: 10.72–42.85, *I*^2^= 98.36%), vomiting (5.99%, 95%CI: 3.65–8.82, *I*^2^ = 93.39%), gastrointestinal reflux (8.58%, 95%CI: 5.51–12.20, *I*^2^ = 93.04%), and food selectivity (59.14%, 95%CI: 44.77–72.76, *I*^2^ = 95.58%). For details please refer to Table 1.

When further conducting the subgroup analysis, we identified an upwards trend in GI rates in ASD in both developed and developing nations. For instance, among developing countries, the GI rate was 40.22% (95% CI: 28.46–52.55) from 2011 to 2020 and 62.31% (95% CI: 27.40–91.32) from 2021 to 2022. In contrast, the prevalence of ASD exhibited an increasing tendency in developed countries and a decreasing trend in developing countries. This is provided in Table 2.

**Table 2:** Time trends in prevalence by level of development.

	**Pooled prevalence**	**95%CI**	**No. cases**	**No. sample**	**No. study**
**Autism spectrum disorders**
**2001–2010**
Developed countries	0.0055	0.0046–0.0066	332,674	62,731,510	8
**2011–2020**
Developed countries	0.0081	0.0073–0.0090	122,057	417,342,043	39
Developing countries	0.0198	0.0129–0.0280	14,539	2,076,225	10
**2021–2022**
Developed countries	0.0138	0.0103–0.0178	732,457	65,532,604	6
Developing countries	0.0092	0.0041–0.0162	4,755	731,366	4
**Gastrointestinal symptoms**
**2001–2010**
Developed countries	0.3830	0.2624–0.5112	590	1,789	11
**2011–2020**
Developed countries	0.4557	0.3729–0.5398	11,700	38,857	26
Developing countries	0.4022	0.2846–0.5255	296	899	9
**2021–2022**
Developed countries	0.6418	0.5729–0.7079	44,919	89,410	12
Developing countries	0.6231	0.2740–0.9132	206	461	5

95%CI=95% Confidence Interval.

## Discussion

The current meta-analysis, based on 126 studies, examined the prevalence of ASD and GI in ASD. We discovered that the global pooled prevalence of ASD was 98 per 10,000, with 48.67% of patients with ASD experiencing at least one GI symptom. ASD is more common in males than females. Both ASD and GI rates increase with time. The overall prevalence of ASD is lower in developed regions than in developing regions.

We found a pooled prevalence of 98/10,000 for ASD, and a recent systematic review (35) reported a median prevalence of 100/10,000 for ASD, which was generally consistent with our results. However, their data are from PubMed only, and the literature did not include a quantitative pooled analysis. In our research, we expanded the search to include additional sources of evidence and performed a quantitative analysis. Furthermore, we made more additions, such as the level of regional development, diagnostic methods, year, and type of study. We discovered some significant differences in total ASD prevalence in subgroup studies.

For both sexes, the prevalence was much higher in men than in women. This was consistent with recent studies (36, 37). This shows a significant male bias in ASD prevalence (38). The reasons for sex disparities in ASD prevalence are still under investigation. A multifactorial paradigm is likely at work, in which risk genes and environmental factors interact with distinct hormonal or immunological pathways according to sex, ultimately contributing to male bias in ASD (39, 40). Another possibility is that some girls may have milder and indistinguishable indications of symptoms that are currently undetectable by diagnostic equipment (41). We additionally found that the prevalence is higher in underdeveloped countries than in developed countries. This emphasizes the role of economics in the prevalence of ASD, possibly because developed areas have a higher quality of life, some of the drivers of ASD are suppressed, more resources are available to limit the risk factors for ASD, and there is greater access to high levels of medical coverage (42). We also took into account regional differences in prevalence. We found that the prevalence was much higher in the Americas than in Asia and Europe. Variances in research methodology, updated diagnosis criteria, and screening tools in national studies may explain regional differences in ASD prevalence (43). We found that the prevalence of ASD increased over time. With the spread of science and medicine, social awareness of ASD has increased. The significant improvement in early identification of the condition explains to some extent the increase in prevalence over time, but this does not imply an increase in the number of cases. The same temporal trend was found in a Korean study (44). Temporal trends in prevalence may reflect differences in exposure to environmental risk factors (45). A further hypothesis is that the environment, which varies in susceptibility over time and at various stages of maternal gestation, may drive the onset of ASD.

We found a pooled prevalence of 48.67% GI problems in autistic patients. The prevalence of GI problems in children with ASD has been reported to be 9%-70%, ranging from mild GI reflux to more severe symptoms (46). GI symptoms show a tendency to recur and may be influenced by a variety of factors. Differences in patient gut flora, a high prevalence of antibiotic use, stress response (47), specific diets, and/or recurrent feeding practices may influence the problem (48).

It is generally known that GI problems can express themselves in a variety of simple or complex ways. We found food selectivity to be one of the most prevalent symptoms in people with ASD. Food selectivity in autistic children frequently includes a strong preference for carbs, snacks, and/or processed meals, as well as a rejection of fruits and vegetables (49). These dietary problems can lead to the development of nutritional deficiencies that can worsen autism symptoms. Food selectivity associated with ASD has been reported to improve with age (50). This may imply that we should provide a wide nutritional supplement rather than a specialized recipe based on dietary preferences. In our study, we also found that another of the most common gastrointestinal symptoms was constipation (26.17%). In a previous review, the median prevalence of constipation was reported to be 22% (51). Most ASD patients have functional constipation associated with mitochondrial dysfunction, gut flora composition, and unusual dietary patterns (52). A 2012 study found that a gluten-free, casein-free (GFCF) diet may be useful in improving behavioral outcomes in children with constipation compared to children with ASD without bowel-related symptoms (53). However, there is a different view that dietary gluten and casein do not correlate with GI symptoms in people with ASD (47). This suggests that the relationship between diet and GI symptoms in ASD is unclear and needs to be further explored in the future. Our results showed that other major gastrointestinal symptoms included abdominal pain, diarrhea, bloating, and nausea. Notably, gastrointestinal issues can manifest in multiple ways. According to Chaidez et al. (54), diarrhea and constipation rarely occur together. 9 children with ASD experienced both diarrhea and constipation in the last 3 months, while 29 children with ASD reported diarrhea along with other symptoms.

The pooled prevalence of Gl problems increased in both developed and developing countries. From 2021 to 2022, both groups had a GI pooled prevalence of ~60%. GI symptoms persist over time as the individual with ASD ages (55). This recurrence may partly explain the increased prevalence of GI. We have also discovered that the prevalence of ASD in wealthy nations has tended to grow year on year, whereas the prevalence in underdeveloped countries has dropped. This may be attributed to the fact that economically developed countries have broader access to medical resources than other locations, leading to the diagnosis of ASD at the start of their development (56), which reflects a trend toward maturity in the available diagnostic procedures. In addition, we hypothesize that the prevalence of health insurance may be higher in developed regions and that parents and providers are willing to consider an ASD diagnosis (57), thus resulting in an increase in the prevalence of ASD.

COVID-19 may impact ASD. The virus may or may not affect the prevalence of ASD, but it may cause delays in diagnosis, treatment, and assistance (58). There is a clear tendency for GI symptoms to spread under the influence of COVID-19 (59), which may also affect the GI in ASD. As a result, we think COVID-19 may act as a genetic, physiological, and pathological driver of ASD. This suggests that more studies with larger sample sizes are needed in the future to investigate the potential link between COVID-19 and ASD.

## Strengths and limitations

Additional databases were searched, and more sources of evidence were included. No restrictions were placed on age in this study, and as many areas as possible were included. Following the COVID-19 epidemic, this research re-examined the prevalence of ASD and conducted the first meta-analysis of the prevalence of GI symptoms in ASD. Our subgroup observed a strong sex difference in ASD and an overall rising tendency in both illnesses. Our findings help to update health policy to better prevent and respond to ASD and associated comorbidities.

Our study also has some limitations. First, the research is unevenly distributed regionally. Relatively more research has been carried out in the Americas, Europe, and Asia and very little in Africa and Oceania. These few pieces of literature may have some influence on the findings for some subgroups, causing some bias, but for the overall rate, the effect is small due to the large sample size. We did not collect data from Chinese databases, which may have caused some linguistic bias, but we included some data from Chinese patients in the published English studies. Finally, GI symptoms are primarily diagnosed by parental reports and symptom questionnaires, and their diagnostic criteria need to be clarified. A more standardized and consistent diagnostic scale for the assessment of GI in patients with ASD is needed in the future.

## Conclusion

We discovered an overall prevalence of 98 per 10,000 for ASD and a high prevalence of 48.67% for GI in ASD, both increasing over time. We found a higher prevalence of ASD in men than in women and a higher prevalence of ASD in developing countries. We should be aware of subtle behavioral and mental changes and the onset of specific gastrointestinal symptoms early in life. Simultaneously, changing policies may help avoid ASD and comorbidities. Due to the high prevalence of ASD and GI symptoms, we will need to continue focusing on this area in the future.

## Data availability statement

The original contributions presented in the study are included in the article/Supplementary material, further inquiries can be directed to the corresponding author/s.

## Author contributions

JyW served as principal author and had full access to all the data in the study, takes responsibility for the accuracy of the data analysis, the integrity of the data, and contributed to draft of the manuscript. ZyZ and JjW contributed to the conception and design. JjW and BM contributed to data acquisition and interpretation. OC contributed to revise of the article and final approval. All authors contributed to the article and approved the submitted version.

## Funding

This research was supported by the Natural Science Foundation of Shandong Province (No. ZR2020MH006).

## Conflict of interest

The authors declare that the research was conducted in the absence of any commercial or financial relationships that could be construed as a potential conflict of interest.

## Publisher's note

All claims expressed in this article are solely those of the authors and do not necessarily represent those of their affiliated organizations, or those of the publisher, the editors and the reviewers. Any product that may be evaluated in this article, or claim that may be made by its manufacturer, is not guaranteed or endorsed by the publisher.
