# Supplementary figures and images for: Global prevalence of autism spectrum disorder and its gastrointestinal symptoms: A systematic review and meta-analysis
Source: Front Psychiatry. 2022 Aug 23;13:963102. doi: 10.3389/fpsyt.2022.963102 (PMC9445193; doi:10.3389/fpsyt.2022.963102)

Funnel plot with pseudo 95% confidence limits

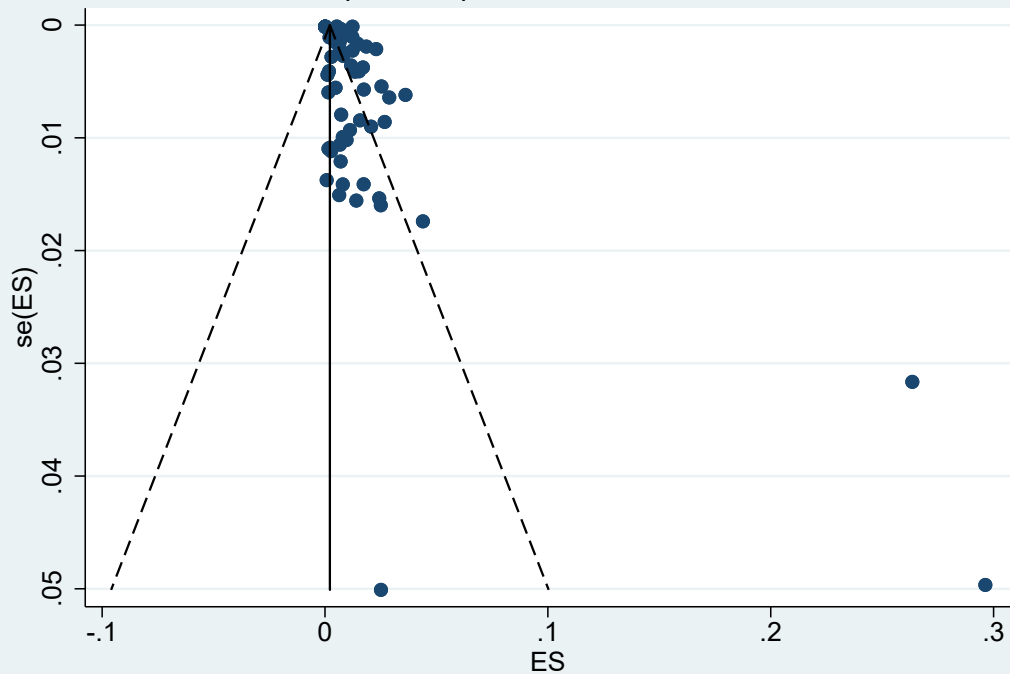

Supplement: Supplementary file 1 [file Data_Sheet_1.PDF]

Funnel plot with pseudo 95% confidence limits

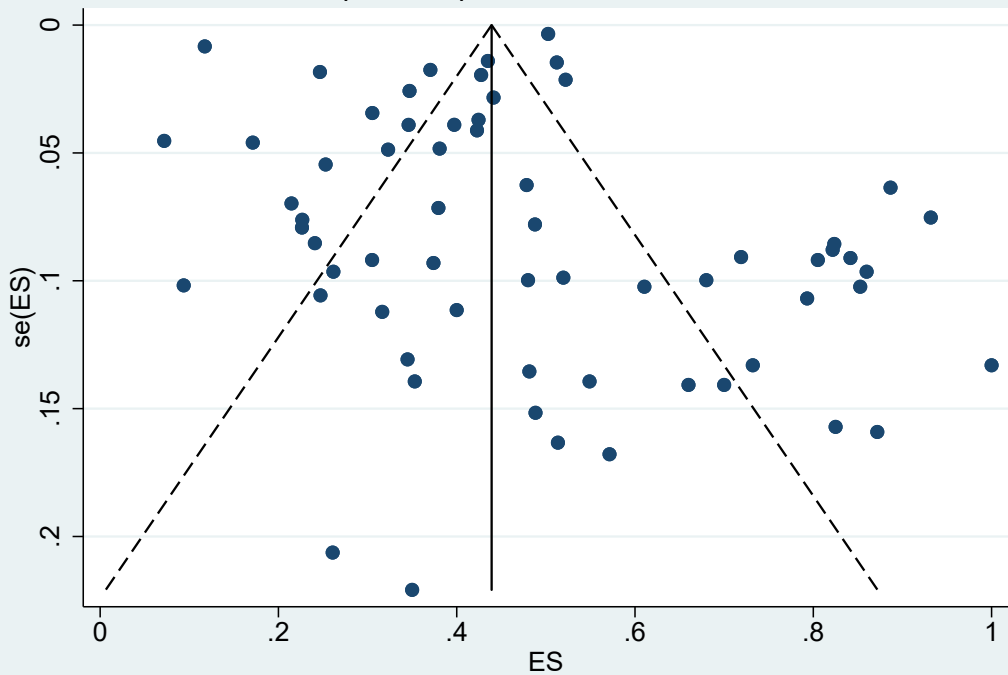

Supplement: Supplementary file 2 [file Data_Sheet_2.PDF]
