# Supplementary material for: Global prevalence of autism spectrum disorder and its gastrointestinal symptoms: A systematic review and meta-analysis
Source: Front Psychiatry. 2022 Aug 23;13:963102. doi: 10.3389/fpsyt.2022.963102 (PMC9445193; doi:10.3389/fpsyt.2022.963102)
Supplement: Supplementary file 3 [file Table_1.DOCX]

1.Literature characteristics of ASD prevalence

| Study | Study design | Age, mean±sd(range) | Proportion of males(%) | Region | Sample | Cases | Diagnostic  criteria | Quality |
| --- | --- | --- | --- | --- | --- | --- | --- | --- |
| Bertrand et al. 2001 | Cross-sectional | 3-10 | 49.06% | United States | 8,896 | 60 | DSM-IV | 6 |
| Gurney et al. 2006 | Cross-sectional | 3-17 | 51.15% | United States | 61,424,000 | 324,000 | Parent-reported | 7 |
| Guiomar et al. 2007 |  |  |  |  |  |  |  |  |
| Cross-sectional _a_ | Cross-sectional | 6-9 | NR | Portugal | 59,478 | 107 | DSM-IV | 10 |
| Cross-sectional _b_ | Cross-sectional | 6-9 | NR | Portugal | 8,317 | 13 | DSM-IV | 10 |
| Catherine et al. 2007 | Cross-sectional | 8 | NR | United States | 187,761 | 1,252 | DSM-IV | 8 |
| Erik et al. 2008 | Cohort study | 7-12 | NR | Denmark | 407,458 | 2,649 | ICD-10 | 8 |
| Michael et al. 2009 | Cross-sectional | 3-17 | NR | United States | 77,911 | 913 | Parent-reported | 7 |
| Durkin et al. 2010 | Cross-sectional | 8 | NR | United States | 557,689 | 3,680 | DSM-IV | 11 |
| Pål et al. 2012 | Cross-sectional | 0-10 | 51.19% | Norway | 731,318 | 2,352 | DSM-IV | 8 |
| Nygren et al. 2012 | Cross-sectional | 2 | NR | Sweden | 5,007 | 40 | DSM-IV | 8 |
| Hsu et al. 2012 | Cross-sectional | 5-27 | 47.60% | China | 884,771 | 10,868 | ICD-9 | 10 |
| Andersen et al. 2013 | Cohort study | 1-16 | NR | Denmark | 1,005,330 | 8,087 | ICD-8  ICD-10 | 11 |
| Michael et al. 2013 | Cross-sectional | 0-12 | 51.49% | Israel | 423,524 | 2,034 | DSM-IV | 10 |
| Phillips et al. 2014 | Cross-sectional | 12-17 | NR | United States | 9,619 | 93 | Parent-reported | 9 |
| Huang et al. 2014 | Cross-sectional | 18-36months | 51.78% | China | 8,000 | 22 | DSM-IV | 8 |
| Jon et al. 2014 | Cross-sectional | 8 | NR | United States | 363,749 | 5,338 | DSM-IV | 8 |
| Zahorodny et al. 2014 | Cross-sectional | 8 | 51.49% | United States | 30,570 | 533 | DSM-IV | 8 |
| Dekkers et al. 2015 | Cross-sectional | 5-15 | NR | Quito | 51,453 | 57 | DSM-III/DSM-IV | 11 |
| Amy et al. 2016 | Cross-sectional | 7-9 | NR | United States | 12,329 | 255 | DSM-IV | 9 |
| Deborah et al. 2018 | Cross-sectional | 8 | NR | United States | 346,978 | 5,021 | DSM-IV | 8 |
| Mpaka et al. 2016 | Cross-sectional | 3-17 | 60.00% | Congo | 405 | 120 | DSM-IV  ADI-R | 8 |
| Melinda et al. 2016 |  |  |  |  |  |  |  |  |
| Cohort _1_ | Cohort study | 6-7 | NR | Australia | 4,235 | 103 | Parent-reported | 10 |
| Cohort _2_ | Cohort study | 6-7 | NR | Australia | 4,127 | 58 | Parent-reported | 9 |
| Monique et al. 2016 | Cross-sectional | 18-30months | NR | Lebanon | 998 | 263 | M-CHAT | 5 |
| Sunil et al. 2017 | Cross-sectional | 1-10 | NR | India | 28,070 | 43 | HISSA | 11 |
| Supekar et al. 2017 | Cross-sectional | 0-35 | NR | United States | 1,847,365 | 4,790 | ICD-9 | 8 |
| Shaheen et al. 2018 | Cross-sectional | 18-36months | NR | Bangladesh | 5,286 | 4 | M-CHAT  DSM- IV  ADOS  Flash card | 8 |
| Narzisi et al. 2018 | Cross-sectional | 7-9 | 51.60% | Italia | 10,138 | 81 | ADOS-2  ADI-R | 8 |
| Tybor et al. 2019 | Cross-sectional | 0-17 | NR | United States | 24,251 | 699 | Parent-reported | 9 |
| Alshaban et al. 2019 | Cross-sectional | 6-11 | NR | Qatar | 133,781 | 1,094 | DSM-5 | 8 |
| Al-Mamri et al. 2019 | Cross-sectional | 0-14 | 50.91% | Oman | 837,655 | 1,705 | DSM-5 | 8 |
| Christensen et al. 2019 |  |  |  |  |  |  |  |  |
| Cross-sectional _a_ | Cross-sectional | 4 | NR | United States | 58,467 | 783 | DSM-IV  DSM-5 | 8 |
| Cross-sectional _b_ | Cross-sectional | 4 | NR | United States | 59,456 | 907 | DSM-IV  DSM-5 | 8 |
| Cross-sectional _c_ | Cross-sectional | 4 | NR | United States | 70,887 | 1,208 | DSM-IV  DSM-5 | 8 |
| Jussila et al. 2020 | Cross-sectional | 8 | 49.28% | Finland | 4,397 | 28 | ASSQ  ADI-R  ADOS | 10 |
| Zablotsky et al. 2020 | Cross-sectional | 3-17 | NR | United States | 33,775 | 856 | Parent-reported | 11 |
| Dickerson et al. 2020 |  |  |  |  |  |  |  |  |
| Cross-sectional _a_ | Cross-sectional | NR | NR | United States | 4,266,489 | 35,555 | DSM-IV | 9 |
| Cross-sectional _b_ | Cross-sectional | NR | NR | United States | 11,524 | 129 | DSM-IV | 9 |
| Saito et al. 2020 | Cross-sectional | 5 | 50.82% | Japan | 5,016 | 87 | DSM-5 | 8 |
| Hong et al. 2020 |  |  |  |  |  |  |  |  |
| Cross-sectional _a_ | Cross-sectional | 0-89 | 50.11% | Korea | 49,540,367 | 2,499 | ICD-10 | 8 |
| Cross-sectional _b_ | Cross-sectional | 0-89 | 50.09% | Korea | 49,773,145 | 2,677 | ICD-10 | 8 |
| Cross-sectional _c_ | Cross-sectional | 0-89 | 50.10% | Korea | 50,515,666 | 3,164 | ICD-10 | 8 |
| Cross-sectional _d_ | Cross-sectional | 0-89 | 50.08% | Korea | 50,734,284 | 3,638 | ICD-10 | 8 |
| Cross-sectional _e_ | Cross-sectional | 0-89 | 50.06% | Korea | 50,948,272 | 4,159 | ICD-10 | 8 |
| Cross-sectional _f_ | Cross-sectional | 0-89 | 50.03% | Korea | 51,141,463 | 4,690 | ICD-10 | 8 |
| Cross-sectional _g_ | Cross-sectional | 0-89 | 50.01% | Korea | 51,327,916 | 5,097 | ICD-10 | 8 |
| Cross-sectional _h_ | Cross-sectional | 0-89 | 49.99% | Korea | 51,529,338 | 5,653 | ICD-10 | 8 |
| May et al. 2020 |  |  |  |  |  |  |  |  |
| Cohort _1_ | Cohort study | 12-13 | NR | Australia | 3,300 | 145 | Parent-reported | 11 |
| Cohort _2_ | Cohort study | 12-13 | NR | Australia | 3,913 | 98 | Parent-reported | 11 |
| Maenner et al. 2020 | Cross-sectional | 8 | NR | United States | 275,419 | 5,108 | DSM-IV  DSM-5 | 8 |
| Zhou et al. 2020 | Cross-sectional | 7-11 | 53.01% | China | 125,806 | 363 | DSM-5 | 8 |
| Magen et al. 2020 | Cohort study | 0-16 | NR | Israel | 1,786,194 | 11,699 | DSM-IV  DSM-5 | 12 |
| Delobel et al. 2020 |  |  |  |  |  |  |  |  |
| Cohort _1_ | Cohort study | 7-9 | 51.37% | Denmark | 195,293 | 2,414 | ICD-10 | 8 |
| Cohort _2_ | Cohort study | 7-9 | 51.14% | Finland | 177,193 | 1,347 | ICD-10 | 8 |
| Cohort _3_ | Cohort study | 7-9 | 51.23% | SW-France | 15,836 | 115 | ICD-10 | 8 |
| Cohort _4_ | Cohort study | 7-9 | 51.31% | SE-France | 32,342 | 154 | ICD-10 | 8 |
| Cohort _5_ | Cohort study | 7-9 | 51.53% | Iceland | 13,551 | 363 | ICD-10 | 8 |
| Thuc et al. 2021 | Cross-sectional | 12-72months | 56.64% | Vietnam | 14,000 | 220 | Medical records | 8 |
| Bosch et al. 2021 | Cross-sectional | 5-17 | 56.37% | Spanish | 6,834 | 48 | Specialist-diagnosed  The K-SADS/PL | 8 |
| Safer et al. 2021 | Cross-sectional | 6-21 | NR | United States | 50,693,053 | 625,215 | DSM-5  DSM | 8 |
| Russell et al. 2021 | Cohort study | ALL | NR | England | 9,597,002 | 65,665 | Diagnostic codes | 8 |
| Maenner et al. 2021 | Cross-sectional | 8 | NR | United States | 220,281 | 5,058 | ICD-9/ICD-10 | 11 |
| Lee et al. 2021 | Cohort study | 0-7 | NR | China | 708,517 | 4,506 | ICD-9 | 11 |
| Arun et al. 2022 | Cross-sectional | 1.5-10 | 52.83% | India | 8,451 | 19 | DSM-5 | 8 |
| Shenouda et al. 2022 | Cross-sectional | 8 | 50.78% | United States | 26,083 | 942 | DSM-5 | 11 |
| Yoo et al. 2022 | Cohort study | 0-8 | 51.69% | Korea | 4,989,351 | 35,529 | ICD-10 | 11 |
| AlBatti et al. 2022 | Cross-sectional | 2-4 | 51.76% | Saudi Arabia | 398 | 10 | M-CHAT  ADOS-2 | 8 |
| Notes：There were multiple sets of data in one study, and subscripts A and B were used in the cross-sectional study.  Subscripts 1 and 2 were used in the cohort study. | | | | | | | | |
| abbreviation ：①NR=Not Reported ②DSM=Diagnostic and Statistical Manual of Mental Disorders ③ICD=International Classification of Diseases  ④ ADIR=Autism Diagnostic Interview Revised ⑤ADOS=Autism Diagnostic Observation Schedule ⑥M-CHAT=Modified Checklist for Autism in Toddlers ⑦ HISSA=Hindi version Indian Scale for Assessment of Autism ⑧ASSQ= Autism Spectrum Screening Questionnaire ⑨K-SADS/PL=Kiddie Schedule for Affective Disorders and Schizophrenia Present and Lifetime version | | | | | | | | |

2.Literature characteristics of GI prevalence in ASD

| Study | Study design | Age, mean±sd(range) | Region | Sample | Cases | Symptom | Quality |
| --- | --- | --- | --- | --- | --- | --- | --- |
| Doenyas et al. 2021 | Cross-sectional | 1-18 | Turkey | 911 | NR | ①⑧ | 11 |
| Koceski et al. 2021 | Case-control | 3-24 | Macedonia | 72 | NR | ①⑦ | 3 |
| Garrick et al. 2021 | Cross-sectional | 0-18 | Australia | 421 | 136 | NR | 10 |
| Dooley et al. 2021 | Cross-sectional | 3-17 | Ireland | 118 | 95 | ①②③④⑤ | 10 |
| Black et al. 2002 | Case-control | 4 | England | 96 | 9 | NR | 7 |
| Taylor et al. 2002 | Cohort study | 0-19 | England | 473 | 81 | ①② | 11 |
| Molloy et al. 2003 | Cross-sectional | 24-96months | United States | 137 | 33 | ①②③④⑥ | 10 |
| Whiteley et al. 2004 | Cross-sectional | 4-6 | England | 512 | NR | ①② | 10 |
| Kerwin et al. 2005 | Cross-sectional | 3--17 | United States | 89 | NR | ③④ | 9 |
| Galli et al. 2006 | Case-control | 19-72 | Switzerland | 43 | 21 | ①②⑤⑦ | 7 |
| Valicenti et al. 2006 | Case-control | 1-18 | United States | 50 | 35 | ①③⑥⑦⑧ | 8 |
| Maria et al. 2006 | Cross-sectional | 1-18 | United States | 100 | 68 | ①③⑥⑦⑧ | 10 |
| Ibrahim et al. 2009 | Case-control | 0-21 | United States | 121 | 87 | ①②⑦⑧ | 6 |
| Smith et al. 2009 | Case-control | 9.7±3.7 | United States | 51 | 18 | ①②③④⑥ | 5 |
| Nikolov et al. 2009 | Cross-sectional | 5-17 | United States | 172 | 39 | ①②⑥⑦ | 9 |
| Campbell et al. 2009 | Cross-sectional | NR | United States | 428 | 163 | NR | 10 |
| Mouridsen et al. 2010 | Case-control | 27-30 | Denmark | 118 | 36 | NR | 8 |
| Wang et al. 2011 | Case-control | 1-18 | United States | 589 | 249 | ①②③⑦ | 8 |
| Russo et al. 2011 | Case-control | 38 | United States | 79 | 25 | ①⑦ | 4 |
| Kohane et al. 2012 | Cross-sectional | 0-34 | United States | 14,381 | 1,688 | NR | 11 |
| Maenner et al. 2012 | Cross-sectional | 8 | United States | 487 | 35 | NR | 10 |
| Gondalia et al. 2012 | Case-control | 2-12 | Australia | 51 | 28 | ①②③④ | 8 |
| Geier et al. 2012 | Cross-sectional | 2-16 | United States | 54 | 26 | ①② | 9 |
| Mouridsen et al. 2013 | Case-control | ALL | Denmark | 89 | 22 | NR | 7 |
| Mannion et al. 2013 | Cross-sectional | 3-16 | Ireland | 87 | 69 | ①②③④⑤ | 10 |
| Mazurek et al. 2013 | Cross-sectional | 2-17 | United States | 2,973 | 733 | ①②③④⑤ | 10 |
| Sun et al. 2013 | Case-control | 4-6 | China | 53 | NR | ①②③④⑧ | 7 |
| Lau et al. 2013 | Case-control | 4-12 | United States | 37 | 19 | ①②⑦ | 5 |
| Valicenti et al. 2014 | Case-control | 2-18 | United States | 50 | 33 | NR | 8 |
| Kang et al. 2014 | Cross-sectional | 2-18 | United States | 164 | 80 | ①②④⑦ | 11 |
| Mazefsky et al. 2014 | Cross-sectional | 7-19 | United States | 95 | 58 | ③④ | 10 |
| Peters et al. 2014 | Cross-sectional | 2-17 | United States | 5,076 | 2,208 | NR | 10 |
| Aldinger et al. 2015 |  |  |  |  |  |  |  |
| Cohort _1_ | Cohort study | 4-14 | United States | 728 | 309 | NR | 10 |
| Cohort _2_ | Cohort study | 4-14 | United States | 2,623 | 1,121 | NR | 10 |
| Mostafa et al. 2015 | Case-control | 3-10 | Saudi Arabia | 100 | 48 | NR | 6 |
| Croen et al. 2015 | Case-control | 29±12.2 | United States | 1,507 | 523 | ①②⑦ | 8 |
| Pusponegoro et al. 2015 | Case-control | 2-10 | India | 159 | 36 | NR | 6 |
| Bresnahan et al. 2015 | Cohort study | 18-36months | Nolway | 195 | 74 | ①② | 11 |
| Attlee et al. 2015 | Cross-sectional | 5-16 | United Arab Emirates | 23 | 6 | ①②⑥⑦ | 10 |
| Ghosh et al. 2015 | Cross-sectional | 2-20 | India | 20 | 7 | ①② | 12 |
| Abdelrahman et al. 2015 | Case-control | 3-17 | Egypt | 80 | 32 | NR | 5 |
| Mannion et al. 2016 | Cohort study | 5-19 | Ireland | 56 | 41 | ①②③④⑤ | 10 |
| Fulceri et al. 2016 | Case-control | 1.6-5.9 | Italy | 115 | 43 | ①②③⑤⑥ | 7 |
| Kheirouri et al. 2016 | Case-control | 4-18 | Iran | 35 | 20 | ①③⑥ | 6 |
| Liu et al. 2016 | Case-control | 3.85-6.33 | China | 154 | NR | ①②⑥⑧ | 8 |
| Marler et al. 2016 | Cross-sectional | 6-18 | United States | 82 | NR | ① | 10 |
| Kushak et al. 2016 | Case-control | 18months-18 | United States | 61 | NR | ②③ | 5 |
| Marler et al. 2017 | Cross-sectional | 6-18 | United States | 108 | NR | ① | 10 |
| Li et al. 2017 | Cross-sectional | 3-8 | China | 336 | 85 | ①②③④⑥ | 11 |
| Penzol et al. 2019 | Cross-sectional | 1-53 | Spain | 845 | 258 | ①②③⑥⑦ | 11 |
| Vargason et al. 2019 | Cohort study | 0-15 | United States | 3,253 | 1,205 | ①②③ | 11 |
| Babinska et al. 2020 | Case-control | 2-18 | The Slovak Republic | 247 | 219 | ①②③④ | 8 |
| Lai et al. 2020 | Case-control | 4-18 | China | 107 | 28 | ①⑥ | 5 |
| Restrepo et al. 2020 | Case-control | 2-3.5 | United States | 255 | 122 | ①②③④⑥ | 6 |
| Silva et al. 2020 | Cross-sectional | 3-10 | Brazil | 39 | 34 | NR | 9 |
| Leader et al. 2020 | Cross-sectional | 8.36±4.13 | Ireland | 136 | 112 | ⑧ | 9 |
| Hand et al. 2020 | Case-control | ≥65 | United States | 4,685 | 2,400 | NR | 7 |
| Azouz et al. 2021 | Case-control | 3-12 | Egypt | 40 | 33 | ①②③④ | 5 |
| DaWalt et al. 2021 | Case-control | ≥18 | United States | 2,187 | 1,142 | NR | 7 |
| Wong et al. 2021 | Case-control | 4-11 | China | 69 | NR | ① | 6 |
| Geraldine et al. 2021 | Cross-sectional | 18-69 | Ireland | 107 | 92 | ①②③④⑤ | 10 |
| Angell et al. 2021 | Cohort study | 1-21 | United States | 83,500 | 41,965 | NR | 11 |
| Reynolds et al. 2021 | Case-control | 2-5 | United States | 656 | 227 | ①②③④⑥ | 7 |
| Fields et al. 2021 | Case-control | 2-5 | United States | 1,244 | 549 | ①②⑥ | 7 |
| Chakraborty et al. 2021 | Cross-sectional | 2-7 | United States | 176 | 164 | ①②③④⑥ | 10 |
| Mairéad et al. 2021 | Cross-sectional | 3-17 | Ireland | 120 | 101 | ①②③④⑤ | 9 |
| Kumar et al. 2021 | Cross-sectional | 11.5±5.4 | India | 58 | 20 | ①③⑥ | 7 |
| Gok et al.2021 | Cross-sectional | 3-18 | Turkey | 102 | 53 | ①②③⑥ | 9 |
| Voulgarakis et al. 2021 | Cross-sectional | 7-11 | United States | 657 | 261 | ②⑥⑦ | 10 |
| Sabbagh et al. 2021 | Cross-sectional | 6-12 | Saudi Arabia | 205 | 44 | NR | 9 |
| Hogan et al. 2021 | Cross-sectional | 3-18 | Ireland | 129 | 106 | ①②③④⑤ | 9 |
| Wiggins et al. 2022 | Case-control | 24-68months | United States | 729 | NR | ①②③④⑥⑦ | 7 |
| Karagözlü et al. 2022 | Case-control | 3-18 | Turkey | 56 | 56 | ①②③④⑦ | 6 |
| Leader et al. 2022 | Cross-sectional | 2-18 | Ireland | 95 | 81 | ①②③④⑤ | 9 |
| Chandler et al. 2013 | Case-control | 10-14 | England | 132 | NR | ①②③⑥ | 6 |
| Notes:①constipation②diarrhea③abdominal pain④abdominal distension⑤nausea⑥vomit⑦gastroesophageal reflux⑧Food selectivity  NR=Not Reported | | | | | | |  |
