# Supplementary material for: Global prevalence of autism spectrum disorder and its gastrointestinal symptoms: A systematic review and meta-analysis
Source: Front Psychiatry. 2022 Aug 23;13:963102. doi: 10.3389/fpsyt.2022.963102 (PMC9445193; doi:10.3389/fpsyt.2022.963102)
Supplement: Supplementary file 4 [file Table_2.DOCX]

1. Quality assessment of the literature for ASD prevalence inclusion:Observational Cohort and Cross-Sectional Studies

| ID | Study | Q1 | Q2 | Q3 | Q4 | Q5 | Q6 | Q7 | Q8 | Q9 | Q10 | Q11 | Q12 | Q13 | Q14 | Summary |
| --- | --- | --- | --- | --- | --- | --- | --- | --- | --- | --- | --- | --- | --- | --- | --- | --- |
| 01 | Bertrand et al. 2001 | 1 | 1 | 1 | 1 | 0 | 0 | 0 | 0 | 0 | 0 | 1 | 00 | 1 | 0 | 6 |
| 02 | Gurney et al. 2006 | 1 | 1 | 1 | 1 | 0 | 1 | 0 | 0 | 0 | 0 | 0 | 00 | 1 | 1 | 7 |
| 03 | Guiomar et al. 2007 _a_ | 1 | 1 | 1 | 1 | 1 | 1 | 0 | 0 | 1 | 0 | 1 | 00 | 1 | 1 | 10 |
| 04 | Guiomar et al. 2007 _b_ | 1 | 1 | 1 | 1 | 1 | 1 | 0 | 0 | 1 | 0 | 1 | 00 | 1 | 1 | 10 |
| 05 | Catherine et al. 2007 | 1 | 1 | 1 | 1 | 1 | 0 | 0 | 0 | 0 | 0 | 1 | 00 | 1 | 1 | 8 |
| 06 | Erik et al. 2008 | 1 | 1 | 1 | 1 | 1 | 0 | 0 | 0 | 0 | 0 | 1 | 00 | 1 | 1 | 8 |
| 07 | Michael et al. 2009 | 1 | 1 | 1 | 1 | 1 | 0 | 0 | 0 | 0 | 0 | 0 | 00 | 1 | 1 | 7 |
| 08 | Durkin et al. 2010 | 1 | 1 | 1 | 1 | 1 | 0 | 0 | 1 | 1 | 1 | 1 | 00 | 1 | 1 | 11 |
| 09 | Pål et al. 2012 | 1 | 1 | 1 | 1 | 1 | 0 | 0 | 0 | 0 | 0 | 1 | 00 | 1 | 1 | 8 |
| 10 | Nygren et al. 2012 | 1 | 1 | 1 | 1 | 1 | 0 | 0 | 0 | 0 | 0 | 1 | 00 | 1 | 1 | 8 |
| 11 | Hsu et al. 2012 | 1 | 1 | 1 | 1 | 1 | 0 | 0 | 1 | 1 | 0 | 1 | 00 | 1 | 1 | 10 |
| 12 | Andersen et al. 2013 | 1 | 1 | 1 | 1 | 1 | 1 | 1 | 0 | 1 | 0 | 1 | 00 | 1 | 1 | 11 |
| 13 | Michael et al. 2013 | 1 | 1 | 1 | 1 | 1 | 0 | 0 | 1 | 1 | 0 | 1 | 00 | 1 | 1 | 10 |
| 14 | Phillips et al. 2014 | 1 | 1 | 1 | 1 | 1 | 1 | 0 | 0 | 0 | 00 | 1 | 00 | 1 | 1 | 9 |
| 15 | Huang et al. 2014 | 1 | 1 | 1 | 1 | 1 | 0 | 0 | 0 | 0 | 0 | 1 | 00 | 1 | 1 | 8 |
| 16 | Jon et al. 2014 | 1 | 1 | 1 | 1 | 1 | 0 | 0 | 0 | 0 | 0 | 1 | 00 | 1 | 1 | 8 |
| 17 | Zahorodny et al. 2014 | 1 | 1 | 1 | 1 | 1 | 0 | 0 | 0 | 0 | 0 | 1 | 00 | 1 | 1 | 8 |
| 18 | Dekkers et al. 2015 | 1 | 1 | 1 | 1 | 1 | 1 | 00 | 1 | 1 | 0 | 1 | 00 | 1 | 1 | 11 |
| 19 | Amy et al. 2016 | 1 | 1 | 1 | 1 | 1 | 0 | 0 | 0 | 1 | 0 | 1 | 00 | 1 | 1 | 9 |
| 20 | Deborah et al. 2016 | 1 | 1 | 1 | 1 | 1 | 0 | 0 | 0 | 0 | 0 | 1 | 00 | 1 | 1 | 8 |
| 21 | Mpaka et al. 2018 | 1 | 1 | 1 | 1 | 1 | 0 | 0 | 0 | 0 | 0 | 1 | 00 | 1 | 1 | 8 |
| 22 | Melinda et al. 2016 _1_ | 1 | 1 | 1 | 1 | 1 | 1 | 00 | 1 | 1 | 00 | 0 | 00 | 1 | 1 | 10 |
| 23 | Melinda et al. 2016 _2_ | 1 | 1 | 1 | 1 | 1 | 1 | 00 | 00 | 1 | 00 | 0 | 00 | 1 | 1 | 9 |
| 24 | Monique et al. 2016 | 1 | 1 | 1 | 0 | 1 | 0 | 0 | 0 | 0 | 0 | 0 | 00 | 1 | 0 | 5 |
| 25 | Sunil et al. 2017 | 1 | 1 | 1 | 1 | 1 | 1 | 0 | 1 | 1 | 0 | 1 | 00 | 1 | 1 | 11 |
| 26 | Supekar et al. 2017 | 1 | 1 | 1 | 1 | 1 | 0 | 0 | 0 | 0 | 0 | 1 | 00 | 1 | 1 | 8 |
| 27 | Shaheen et al. 2018 | 1 | 1 | 1 | 1 | 1 | 0 | 0 | 0 | 0 | 0 | 1 | 00 | 1 | 1 | 8 |
| 28 | Narzisi et al. 2018 | 1 | 1 | 1 | 1 | 1 | 0 | 0 | 0 | 0 | 0 | 1 | 00 | 1 | 1 | 8 |
| 29 | Tybor et al. 2019 | 1 | 1 | 1 | 1 | 1 | 00 | 00 | 00 | 1 | 00 | 1 | 00 | 1 | 1 | 9 |
| 30 | Alshaban et al. 2019 | 1 | 1 | 1 | 1 | 1 | 0 | 0 | 0 | 0 | 0 | 1 | 00 | 1 | 1 | 8 |
| 31 | Al-Mamri et al. 2019 | 1 | 1 | 1 | 1 | 1 | 0 | 0 | 0 | 0 | 0 | 1 | 00 | 1 | 1 | 8 |
| 32 | Christensen et al. 2019 _a_ | 1 | 1 | 1 | 1 | 1 | 0 | 0 | 0 | 0 | 0 | 1 | 00 | 1 | 1 | 8 |
| 33 | Christensen et al. 2019 _b_ | 1 | 1 | 1 | 1 | 1 | 0 | 0 | 0 | 0 | 0 | 1 | 00 | 1 | 1 | 8 |
| 34 | Christensen et al. 2019 _c_ | 1 | 1 | 1 | 1 | 1 | 0 | 0 | 0 | 0 | 0 | 1 | 00 | 1 | 1 | 8 |
| 35 | Jussila et al. 2020 | 1 | 1 | 1 | 1 | 1 | 1 | 00 | 00 | 1 | 00 | 1 | 00 | 1 | 1 | 10 |
| 36 | Zablotsky et al. 2020 | 1 | 1 | 1 | 1 | 1 | 1 | 1 | 00 | 1 | 00 | 1 | 00 | 1 | 1 | 11 |
| 37 | Dickerson et al. 2020 _a_ | 1 | 1 | 1 | 1 | 1 | 0 | 0 | 0 | 1 | 00 | 1 | 00 | 1 | 1 | 9 |
| 38 | Dickerson et al. 2020 _b_ | 1 | 1 | 1 | 1 | 1 | 0 | 0 | 0 | 1 | 00 | 1 | 00 | 1 | 1 | 9 |
| 39 | Saito et al. 2020 | 1 | 1 | 1 | 1 | 1 | 0 | 0 | 0 | 0 | 0 | 1 | 00 | 1 | 1 | 8 |
| 40 | Hong et al. 2020 _a_ | 1 | 1 | 1 | 1 | 1 | 0 | 0 | 0 | 0 | 0 | 1 | 00 | 1 | 1 | 8 |
| 41 | Hong et al. 2020 _b_ | 1 | 1 | 1 | 1 | 1 | 0 | 0 | 0 | 0 | 0 | 1 | 00 | 1 | 1 | 8 |
| 42 | Hong et al. 2020 _c_ | 1 | 1 | 1 | 1 | 1 | 0 | 0 | 0 | 0 | 0 | 1 | 00 | 1 | 1 | 8 |
| 43 | Hong et al. 2020 _d_ | 1 | 1 | 1 | 1 | 1 | 0 | 0 | 0 | 0 | 0 | 1 | 00 | 1 | 1 | 8 |
| 44 | Hong et al. 2020 _e_ | 1 | 1 | 1 | 1 | 1 | 0 | 0 | 0 | 0 | 0 | 1 | 00 | 1 | 1 | 8 |
| 45 | Hong et al. 2020 _f_ | 1 | 1 | 1 | 1 | 1 | 0 | 0 | 0 | 0 | 0 | 1 | 00 | 1 | 1 | 8 |
| 46 | Hong et al. 2020 _g_ | 1 | 1 | 1 | 1 | 1 | 0 | 0 | 0 | 0 | 0 | 1 | 00 | 1 | 1 | 8 |
| 47 | Hong et al. 2020 _h_ | 1 | 1 | 1 | 1 | 1 | 0 | 0 | 0 | 0 | 0 | 1 | 00 | 1 | 1 | 8 |
| 48 | May et al. 2020 _1_ | 1 | 1 | 1 | 1 | 1 | 1 | 0 | 1 | 1 | 0 | 1 | 00 | 1 | 1 | 11 |
| 49 | May et al. 2020 _2_ | 1 | 1 | 1 | 1 | 1 | 1 | 0 | 1 | 1 | 0 | 1 | 00 | 1 | 1 | 11 |
| 50 | Maenner et al. 2020 | 1 | 1 | 1 | 1 | 1 | 0 | 0 | 0 | 0 | 0 | 1 | 00 | 1 | 1 | 8 |
| 51 | Zhou et al. 2020 | 1 | 1 | 1 | 1 | 1 | 0 | 0 | 0 | 0 | 0 | 1 | 00 | 1 | 1 | 8 |
| 52 | Magen et al. 2020 | 1 | 1 | 1 | 1 | 1 | 1 | 1 | 0 | 1 | 1 | 1 | 00 | 1 | 1 | 12 |
| 53 | Delobel et al. 2020 _1_ | 1 | 1 | 1 | 1 | 1 | 0 | 0 | 0 | 0 | 0 | 1 | 00 | 1 | 1 | 8 |
| 54 | Delobel et al. 2020 _2_ | 1 | 1 | 1 | 1 | 1 | 0 | 0 | 0 | 0 | 0 | 1 | 00 | 1 | 1 | 8 |
| 55 | Delobel et al. 2020 _3_ | 1 | 1 | 1 | 1 | 1 | 0 | 0 | 0 | 0 | 0 | 1 | 00 | 1 | 1 | 8 |
| 56 | Delobel et al. 2020 _4_ | 1 | 1 | 1 | 1 | 1 | 0 | 0 | 0 | 0 | 0 | 1 | 00 | 1 | 1 | 8 |
| 57 | Delobel et al. 2020 _5_ | 1 | 1 | 1 | 1 | 1 | 0 | 0 | 0 | 0 | 0 | 1 | 00 | 1 | 1 | 8 |
| 58 | Thuc et al. 2021 | 1 | 1 | 1 | 1 | 1 | 0 | 0 | 0 | 0 | 0 | 1 | 00 | 1 | 1 | 8 |
| 59 | Bosch et al. 2021 | 1 | 1 | 1 | 1 | 1 | 0 | 0 | 0 | 0 | 0 | 1 | 00 | 1 | 1 | 8 |
| 60 | Safer et al. 2021 | 1 | 1 | 1 | 1 | 1 | 0 | 0 | 0 | 0 | 0 | 1 | 00 | 1 | 1 | 8 |
| 61 | Russell et al. 2021 | 1 | 1 | 1 | 1 | 1 | 0 | 0 | 0 | 0 | 0 | 1 | 00 | 1 | 1 | 8 |
| 62 | Maenner et al. 2021 | 1 | 1 | 1 | 1 | 1 | 1 | 0 | 1 | 1 | 0 | 1 | 00 | 1 | 1 | 11 |
| 63 | Lee et al. 2021 | 1 | 1 | 1 | 1 | 1 | 1 | 1 | 0 | 1 | 0 | 1 | 00 | 1 | 1 | 11 |
| 64 | Arun et al. 2022 | 1 | 1 | 1 | 1 | 1 | 0 | 0 | 0 | 0 | 0 | 1 | 00 | 1 | 1 | 8 |
| 65 | Shenouda et al. 2022 | 1 | 1 | 1 | 1 | 1 | 1 | 0 | 1 | 1 | 0 | 1 | 00 | 1 | 1 | 11 |
| 66 | Yoo et al. 2022 | 1 | 1 | 1 | 1 | 1 | 1 | 1 | 0 | 1 | 0 | 1 | 00 | 1 | 1 | 11 |
| 67 | AlBatti et al. 2022 | 1 | 1 | 1 | 1 | 1 | 0 | 0 | 0 | 0 | 0 | 1 | 00 | 1 | 1 | 8 |
| Notes:1=Yes;0=No;00=Not Reported;For multiple studies within the same article, subscripts distinguish between cohort studies where the subscript is a number and cross-sectional studies where the subscript is a letter;Q1:Was the research question or objective in this paper clearly stated?Q2: Was the study population clearly specified and defined?Q3:Was the participation rate of eligible persons at least 50%?Q4:Were all the subjects selected or recruited from the same or similar populations (including the same time period)? Were inclusion and exclusion criteria for being in the study prespecified and applied uniformly to all participants?Q5:Was a sample size justification, power description, or variance and effect estimates provided? Q6:For the analyses in this paper, were the exposure(s) of interest measured prior to the outcome(s) being measured?Q7:Was the timeframe sufficient so that one could reasonably expect to see an association between exposure and outcome if it existed?Q8:For exposures that can vary in amount or level, did the study examine different levels of the exposure as related to the outcome (e.g., categories of exposure, or exposure measured as continuous variable)?Q9:Were the exposure measures (independent variables) clearly defined, valid, reliable, and implemented consistently across all study participants?Q10:Was the exposure(s) assessed more than once over time?Q11:Were the outcome measures (dependent variables) clearly defined, valid, reliable, and implemented consistently across all study participants?Q12:Were the outcome assessors blinded to the exposure status of participants?Q13: Was loss to follow-up after baseline 20% or less?Q14:Were key potential confounding variables measured and adjusted statistically for their impact on the relationship between exposure(s) and outcome(s)? | | | | | | | | | | | | | | | | |

1. Quality of included literature on the incidence of GI in patients with ASD
2. Observational Cohort and Cross-Sectional Studies

| ID | Study | Q1 | Q2 | Q3 | Q4 | Q5 | Q6 | Q7 | Q8 | Q9 | Q10 | Q11 | Q12 | Q13 | Q14 | Summary |
| --- | --- | --- | --- | --- | --- | --- | --- | --- | --- | --- | --- | --- | --- | --- | --- | --- |
| 01 | Doenyas et al. 2021 | 1 | 1 | 1 | 1 | 1 | 1 | 0 | 1 | 1 | 0 | 1 | 00 | 1 | 1 | 11 |
| 02 | Garrick et al. 2021 | 1 | 1 | 1 | 1 | 1 | 1 | 0 | 00 | 1 | 0 | 1 | 00 | 1 | 1 | 10 |
| 03 | Dooley et al. 2021 | 1 | 1 | 1 | 1 | 1 | 1 | 0 | 00 | 1 | 0 | 1 | 00 | 1 | 1 | 10 |
| 04 | Taylor et al. 2002 | 1 | 1 | 1 | 1 | 1 | 1 | 1 | 0 | 1 | 0 | 1 | 00 | 1 | 1 | 11 |
| 05 | Molloy et al. 2003 | 1 | 1 | 1 | 1 | 1 | 1 | 0 | 00 | 1 | 0 | 1 | 00 | 1 | 1 | 10 |
| 06 | Whiteley et al. 2004 | 1 | 1 | 1 | 1 | 1 | 1 | 0 | 00 | 1 | 0 | 1 | 00 | 1 | 1 | 10 |
| 07 | Kerwin et al. 2005 | 1 | 1 | 1 | 1 | 1 | 1 | 0 | 00 | 1 | 0 | 1 | 00 | 1 | 0 | 9 |
| 08 | Maria et al. 2006 | 1 | 1 | 1 | 1 | 1 | 1 | 0 | 00 | 1 | 0 | 1 | 00 | 1 | 1 | 10 |
| 09 | Nikolov et al. 2009 | 1 | 1 | 1 | 1 | 1 | 1 | 0 | 00 | 1 | 0 | 1 | 00 | 1 | 0 | 9 |
| 10 | Campbell et al. 2009 | 1 | 1 | 1 | 1 | 1 | 1 | 0 | 0 | 1 | 0 | 1 | 00 | 1 | 1 | 10 |
| 11 | Kohane et al. 2012 | 1 | 1 | 1 | 1 | 1 | 1 | 1 | 00 | 1 | 0 | 1 | 00 | 1 | 1 | 11 |
| 12 | Maenner et al. 2012 | 1 | 1 | 1 | 1 | 1 | 1 | 0 | 00 | 1 | 0 | 1 | 00 | 1 | 1 | 10 |
| 13 | Geier et al. 2012 | 1 | 1 | 1 | 1 | 1 | 1 | 0 | 00 | 1 | 0 | 1 | 00 | 1 | 0 | 9 |
| 14 | Mannion et al. 2013 | 1 | 1 | 1 | 1 | 1 | 1 | 0 | 00 | 1 | 0 | 1 | 00 | 1 | 1 | 10 |
| 15 | Mazurek et al. 2013 | 1 | 1 | 1 | 1 | 1 | 1 | 0 | 00 | 1 | 0 | 1 | 00 | 1 | 1 | 10 |
| 16 | Kang et al. 2014 | 1 | 1 | 1 | 1 | 1 | 1 | 1 | 00 | 1 | 0 | 1 | 00 | 1 | 1 | 11 |
| 17 | Mazefsky et al. 2014 | 1 | 1 | 1 | 1 | 1 | 1 | 0 | 1 | 1 | 0 | 0 | 00 | 1 | 1 | 10 |
| 18 | Peters et al. 2014 | 1 | 1 | 1 | 1 | 1 | 1 | 0 | 00 | 1 | 0 | 1 | 00 | 1 | 1 | 10 |
| 19 | Aldinger et al. 2015 _1_ | 1 | 1 | 1 | 1 | 1 | 1 | 0 | 00 | 1 | 0 | 1 | 00 | 1 | 1 | 10 |
| 20 | Aldinger et al. 2015 _2_ | 1 | 1 | 1 | 1 | 1 | 1 | 0 | 00 | 1 | 0 | 1 | 00 | 1 | 1 | 10 |
| 21 | Bresnahan et al. 2015 | 1 | 1 | 1 | 1 | 1 | 1 | 1 | 00 | 1 | 0 | 1 | 00 | 1 | 1 | 11 |
| 22 | Attlee et al. 2015 | 1 | 1 | 1 | 1 | 1 | 1 | 1 | 00 | 1 | 0 | 1 | 00 | 1 | 0 | 10 |
| 23 | Ghosh et al. 2015 | 1 | 1 | 1 | 1 | 1 | 1 | 1 | 1 | 1 | 0 | 1 | 00 | 1 | 1 | 12 |
| 24 | Mannion et al. 2016 | 1 | 1 | 1 | 1 | 1 | 1 | 1 | 00 | 1 | 0 | 1 | 00 | 1 | 0 | 10 |
| 25 | Marler et al. 2016 | 1 | 1 | 1 | 1 | 1 | 1 | 0 | 00 | 1 | 0 | 1 | 00 | 1 | 1 | 10 |
| 26 | Marler et al. 2017 | 1 | 1 | 1 | 1 | 1 | 1 | 0 | 00 | 1 | 0 | 1 | 00 | 1 | 1 | 10 |
| 27 | Li et al. 2017 | 1 | 1 | 1 | 1 | 1 | 1 | 1 | 00 | 1 | 0 | 1 | 00 | 1 | 1 | 11 |
| 28 | Penzol et al. 2019 | 1 | 1 | 1 | 1 | 1 | 1 | 1 | 00 | 1 | 0 | 1 | 00 | 1 | 1 | 11 |
| 29 | Vargason et al. 2019 | 1 | 1 | 1 | 1 | 1 | 1 | 1 | 00 | 1 | 0 | 1 | 00 | 1 | 1 | 11 |
| 30 | Silva et al. 2020 | 1 | 1 | 1 | 1 | 1 | 1 | 0 | 00 | 0 | 0 | 1 | 00 | 1 | 1 | 9 |
| 31 | Leader et al. 2020 | 1 | 1 | 1 | 1 | 1 | 1 | 0 | 00 | 1 | 0 | 1 | 00 | 1 | 0 | 9 |
| 32 | Geraldine et al. 2021 | 1 | 1 | 1 | 1 | 1 | 1 | 0 | 00 | 1 | 0 | 1 | 00 | 1 | 1 | 10 |
| 33 | Angell et al. 2021 | 1 | 1 | 1 | 1 | 1 | 1 | 1 | 00 | 1 | 0 | 1 | 00 | 1 | 1 | 11 |
| 34 | Chakraborty et al. 2021 | 1 | 1 | 1 | 1 | 1 | 1 | 0 | 00 | 1 | 0 | 1 | 00 | 1 | 1 | 10 |
| 35 | Mairéad et al. 2021 | 1 | 1 | 1 | 1 | 0 | 1 | 0 | 00 | 1 | 0 | 1 | 00 | 1 | 1 | 9 |
| 36 | Kumar et al, 2021 | 1 | 1 | 1 | 1 | 0 | 1 | 0 | 00 | 0 | 0 | 1 | 00 | 1 | 0 | 7 |
| 37 | Gok et al.2021 | 1 | 1 | 1 | 0 | 1 | 1 | 0 | 00 | 1 | 0 | 1 | 00 | 1 | 1 | 9 |
| 38 | Voulgarakis et al. 2021 | 1 | 1 | 1 | 1 | 1 | 1 | 0 | 00 | 1 | 0 | 1 | 00 | 1 | 1 | 10 |
| 39 | Sabbagh et al. 2021 | 1 | 1 | 1 | 1 | 1 | 1 | 0 | 1 | 1 | 0 | 1 | 00 | 0 | 0 | 9 |
| 40 | Hogan et al. 2021 | 1 | 1 | 1 | 1 | 0 | 1 | 0 | 00 | 1 | 0 | 1 | 00 | 1 | 1 | 9 |
| 41 | Leader et al. 2022 | 1 | 1 | 1 | 1 | 0 | 1 | 0 | 00 | 1 | 0 | 1 | 00 | 1 | 1 | 9 |
| Notes:1=Yes;0=No;00=Not Reported;For multiple studies within the same article, subscripts distinguish them, and subscripts with numbers indicate cohort studies;Q1:Was the research question or objective in this paper clearly stated?Q2: Was the study population clearly specified and defined?Q3:Was the participation rate of eligible persons at least 50%?Q4:Were all the subjects selected or recruited from the same or similar populations (including the same time period)? Were inclusion and exclusion criteria for being in the study prespecified and applied uniformly to all participants?Q5:Was a sample size justification, power description, or variance and effect estimates provided? Q6:For the analyses in this paper, were the exposure(s) of interest measured prior to the outcome(s) being measured?Q7:Was the timeframe sufficient so that one could reasonably expect to see an association between exposure and outcome if it existed?Q8:For exposures that can vary in amount or level, did the study examine different levels of the exposure as related to the outcome (e.g., categories of exposure, or exposure measured as continuous variable)?Q9:Were the exposure measures (independent variables) clearly defined, valid, reliable, and implemented consistently across all study participants?Q10:Was the exposure(s) assessed more than once over time?Q11:Were the outcome measures (dependent variables) clearly defined, valid, reliable, and implemented consistently across all study participants?Q12:Were the outcome assessors blinded to the exposure status of participants?Q13:Was loss to follow-up after baseline 20% or less?Q14:Were key potential confounding variables measured and adjusted statistically for their impact on the relationship between exposure(s) and outcome(s)? | | | | | | | | | | | | | | | | |

1. Case-Control Studies

| ID | Study | Q1 | Q2 | Q3 | Q4 | Q5 | Q6 | Q7 | Q8 | Q9 | Q10 | Q11 | Q12 | Summary |
| --- | --- | --- | --- | --- | --- | --- | --- | --- | --- | --- | --- | --- | --- | --- |
| 01 | Koceski et al. 2021 | 0 | 0 | 0 | 1 | 0 | 1 | 00 | 0 | 0 | 1 | 00 | 0 | 3 |
| 02 | Black et al. 2002 | 1 | 1 | 0 | 1 | 1 | 1 | 00 | 0 | 1 | 1 | 00 | 0 | 7 |
| 03 | Galli et al. 2006 | 1 | 1 | 0 | 1 | 1 | 1 | 00 | 0 | 0 | 1 | 00 | 1 | 7 |
| 04 | Valicenti et al. 2006 | 1 | 1 | 1 | 1 | 1 | 1 | 00 | 0 | 0 | 1 | 00 | 1 | 8 |
| 05 | Ibrahim et al. 2009 | 1 | 1 | 0 | 1 | 1 | 1 | 00 | 0 | 0 | 1 | 00 | 0 | 6 |
| 06 | Smith et al. 2009 | 1 | 0 | 1 | 0 | 1 | 1 | 00 | 0 | 0 | 1 | 00 | 0 | 5 |
| 07 | Mouridsen et al. 2010 | 1 | 1 | 1 | 1 | 1 | 1 | 00 | 0 | 0 | 1 | 00 | 1 | 8 |
| 08 | Wang et al. 2011 | 1 | 1 | 1 | 1 | 1 | 1 | 00 | 0 | 0 | 1 | 00 | 1 | 8 |
| 09 | Russo et al. 2011 | 0 | 0 | 0 | 0 | 1 | 1 | 00 | 0 | 1 | 1 | 00 | 0 | 4 |
| 10 | Gondalia et al. 2012 | 1 | 1 | 0 | 1 | 1 | 1 | 00 | 0 | 1 | 1 | 00 | 1 | 8 |
| 11 | Mouridsen et al. 2013 | 0 | 1 | 1 | 1 | 1 | 1 | 00 | 0 | 0 | 1 | 00 | 1 | 7 |
| 12 | Sun et al. 2013 | 1 | 1 | 1 | 1 | 1 | 1 | 00 | 0 | 0 | 1 | 00 | 0 | 7 |
| 13 | Lau et al. 2013 | 0 | 0 | 1 | 0 | 1 | 1 | 00 | 0 | 00 | 1 | 00 | 1 | 5 |
| 14 | Valicenti et al. 2014 | 1 | 1 | 1 | 1 | 1 | 1 | 00 | 0 | 0 | 1 | 00 | 1 | 8 |
| 15 | Mostafa et al. 2015 | 1 | 0 | 1 | 1 | 1 | 1 | 00 | 0 | 0 | 1 | 00 | 0 | 6 |
| 16 | Croen et al. 2015 | 0 | 1 | 1 | 1 | 1 | 1 | 1 | 0 | 0 | 1 | 00 | 1 | 8 |
| 17 | Pusponegoro et al. 2015 | 0 | 1 | 1 | 1 | 1 | 1 | 00 | 0 | 0 | 1 | 00 | 0 | 6 |
| 18 | Abdelrahman et al. 2015 | 1 | 0 | 1 | 00 | 1 | 1 | 00 | 0 | 0 | 1 | 00 | 0 | 5 |
| 19 | Fulceri et al. 2016 | 1 | 1 | 1 | 1 | 1 | 1 | 00 | 0 | 0 | 1 | 00 | 0 | 7 |
| 20 | Kheirouri et al. 2016 | 0 | 0 | 1 | 1 | 1 | 1 | 00 | 0 | 0 | 1 | 00 | 1 | 6 |
| 21 | Liu et al. 2016 | 1 | 1 | 1 | 1 | 1 | 1 | 00 | 0 | 0 | 1 | 00 | 1 | 8 |
| 22 | Kushak et al. 2016 | 1 | 0 | 1 | 00 | 1 | 1 | 00 | 0 | 0 | 1 | 00 | 0 | 5 |
| 23 | Babinska et al. 2020 | 1 | 1 | 1 | 1 | 1 | 1 | 00 | 0 | 0 | 1 | 00 | 1 | 8 |
| 24 | Lai et al. 2020 | 1 | 0 | 0 | 1 | 1 | 1 | 00 | 0 | 0 | 1 | 00 | 0 | 5 |
| 25 | Restrepo et al. 2020 | 1 | 0 | 1 | 00 | 1 | 1 | 00 | 0 | 0 | 1 | 00 | 1 | 6 |
| 26 | Hand et al. 2020 | 1 | 1 | 0 | 1 | 1 | 1 | 00 | 0 | 0 | 1 | 00 | 1 | 7 |
| 27 | Azouz et al. 2021 | 1 | 0 | 1 | 00 | 1 | 1 | 00 | 0 | 0 | 1 | 00 | 0 | 5 |
| 28 | DaWalt et al. 2021 | 1 | 1 | 0 | 1 | 1 | 1 | 00 | 0 | 0 | 1 | 00 | 1 | 7 |
| 29 | Wong et al. 2021 | 1 | 0 | 0 | 1 | 1 | 1 | 00 | 0 | 0 | 1 | 00 | 1 | 6 |
| 30 | Reynolds et al. 2021 | 1 | 0 | 1 | 1 | 1 | 1 | 1 | 0 | 0 | 0 | 00 | 1 | 7 |
| 31 | Fields et al. 2021 | 1 | 1 | 0 | 1 | 0 | 1 | 1 | 0 | 0 | 1 | 00 | 1 | 7 |
| 32 | Wiggins et al. 2022 | 1 | 1 | 0 | 1 | 1 | 1 | 00 | 0 | 0 | 1 | 00 | 1 | 7 |
| 33 | Karagözlü et al. 2022 | 1 | 1 | 1 | 00 | 1 | 1 | 00 | 0 | 0 | 1 | 00 | 0 | 6 |
| 34 | Chandler et al. 2013 | 0 | 1 | 0 | 1 | 1 | 1 | 00 | 0 | 0 | 1 | 00 | 1 | 6 |
| Notes:1=Yes;0=No;00=Not Reported;Q1:Was the research question or objective in this paper clearly stated and appropriate?Q2:Was the study population clearly specified and defined?Q3: Did the authors include a sample size justification?Q4:Were controls selected or recruited from the same or similar population that gave rise to the cases (including the same timeframe)?Q5:Were the definitions, inclusion and exclusion criteria, algorithms or processes used to identify or select cases and controls valid, reliable, and implemented consistently across all study participants?Q6:Were the cases clearly defined and differentiated from controls?Q7: If less than 100 percent of eligible cases and/or controls were selected for the study, were the cases and/or controls randomly selected from those eligible?Q8: Was there use of concurrent controls?Q9:Were the investigators able to confirm that the exposure/risk occurred prior to the development of the condition or event that defined a participant as a case?Q10:Were the measures of exposure/risk clearly defined, valid, reliable, and implemented consistently (including the same time period) across all study participants?Q11:Were the assessors of exposure/risk blinded to the case or control status of participants?Q12:Were key potential confounding variables measured and adjusted statistically in the analyses? If matching was used, did the investigators account for matching during study analysis? | | | | | | | | | | | | | | |
